# An anti-perfringolysin O monoclonal antibody cross-reactive with streptolysin O protects against streptococcal toxic shock syndrome

**DOI:** 10.1186/s13104-020-05264-2

**Published:** 2020-09-05

**Authors:** Takayuki Matsumura, Ayae Nishiyama, Michio Aiko, Akira Ainai, Tadayoshi Ikebe, Joe Chiba, Manabu Ato, Yoshimasa Takahashi

**Affiliations:** 1 Department of Immunology, National Institute of Infectious Diseases, 1-23-1 Toyama, Shinjuku-ku, Tokyo, 162-8640 Japan; 2 Department of Pathology, National Institute of Infectious Diseases, 1-23-1 Toyama, Shinjuku-ku, Tokyo, 162-8640 Japan; 3 Department of Bacteriology I, National Institute of Infectious Diseases, 1-23-1 Toyama, Shinjuku-ku, Tokyo, 162-8640 Japan; 4 Department of Mycobacteriology, Leprosy Research Center, National Institute of Infectious Diseases, 4-2-1 Aobacho, Higashimurayamashi, Tokyo 189-0002 Japan

**Keywords:** Streptococcal toxic shock syndrome, Cholesterol-dependent cytolysins, Streptolysin O, Perfringolysin O, Neutralizing monoclonal antibody

## Abstract

**Objective:**

*Streptococcus pyogenes* (Group A *Streptococcus*; GAS) causes a variety of infections that include life-threatening, severe invasive GAS infections, such as streptococcal toxic shock syndrome (STSS), with > 30% mortality rate, despite effective antibiotics and treatment options. STSS clinical isolates highly express streptolysin O (SLO), a member of a large family of pore-forming toxins called cholesterol-dependent cytolysins (CDCs). SLO is an important toxic factor for GAS and may be an effective therapeutic target for the treatment of STSS. Our aim was to identify a monoclonal antibody (mAb) that reacts with SLO and has therapeutic potential for STSS treatment.

**Results:**

We focused on mAbs that had originally been established as neutralizing reagents to perfringolysin O (PFO), another member of the CDC family, as some cross-reactivity with SLO had been reported. Here, we confirmed cross-reactivity of an anti-PFO mAb named HS1 with SLO. In vitro analysis revealed that HS1 mAb sufficiently prevented human neutrophils from being killed by STSS clinical isolates. Furthermore, prophylactic and therapeutic injection of HS1 mAb into C57BL/6 mice significantly improved the survival rate following lethal infection with an STSS clinical isolate. These results highlight the therapeutic potential of HS1 mAb for STSS treatment.

## Introduction

*Streptococcus pyogenes*, also known as group A *Streptococcus* (GAS), is one of the most common human pathogens, and causes a wide spectrum of diseases ranging from pharyngitis and skin lesions to streptococcal toxic shock syndrome (STSS) and necrotizing fasciitis. The first two are generally self-limiting, while the latter two are severe and life-threatening, with mortality rates of 30–70% even with immediate antibiotic therapy and intensive care [1–4]. Therefore, GAS is sometimes referred to as “killer microbes” or “flesh-eating bacteria” [5]. The prevalence of severe GAS disease is at least 18.1 million cases globally each year [6], including annual estimates of 8000–24,000 invasive cases; the numbers have been increasing since 2013 in the United States [7]. In addition, in Japan, there are 90–1000 cases of severe invasive streptococcal infections annually, and the number of cases has been similarly increasing since 2014 [8].

Severe invasive GAS clinical isolates often express virulence factors more abundantly than non-invasive GAS clinical isolates. For example, STSS clinical isolates highly express streptolysin O (SLO), which belongs to a large family of pore-forming toxins called the cholesterol-dependent cytolysins (CDCs) [9–13]. SLO has been shown to be a key virulence factor of GAS by preventing internalization of the bacteria into lysosomes where they can be destroyed [14], and has cytopathic effects on neutrophils, macrophages, and dendritic cells [15–19]. As a result, STSS causes severe neutropenia, which results in poor prognosis for the patients and the mouse model [20, 21]. The global increase in invasive GAS infections in the 1980s was associated with the emergence of an M1T1 clone that harbors a 36-kb pathogenicity island that codes for increased expression of SLO and its cotoxin NAD^+^-glycohydrolase [22–25]. Taken together, SLO is a potential therapeutic target for the treatment of STSS.

Several hybridomas that produce monoclonal antibodies (mAbs) specific for *Clostridium perfringens*-derived perfringolysin O (PFO) in the CDC family [26] have been cryopreserved in liquid nitrogen since 1984. Among the original mAbs (4D8, 2C5, and 3H10), 3H10 mAb was found to neutralize PFO and SLO, and inhibited toxin-induced hemolysis of sheep red blood cells and cardiotoxicity in cultured mouse heart cells [26]. The aim of the present study was to re-clone the existing hybridomas and test the specificity of the mAbs against SLO, in an attempt to identify a novel treatment strategy for STSS.

## Main text

### Methods

#### Blinding at any stage of the study

The experiments were not randomized and the investigators were not blinded to allocation during experiments and outcome assessment.

#### Re-cloning of hybridomas

Hybridomas that produce mAbs (mouse IgG1) reactive with PFO were recovered from liquid nitrogen. 3H10 and 2C5 hybridomas were re-cloned by the limiting dilution method at 0.3 cell/well in a 96-well plate, resulting in 7 and 11 colonies, respectively. 4D8 hybridoma was re-cloned at 1 cell/well in a 96-well plate, resulting in 5 colonies. The re-cloned hybridomas 3H10, 4D8, and 2C5 that showed the most potent binding activities against PFO were renamed HS1, HS2, and HS3, respectively.

#### Enzyme-linked immunosorbent assay (ELISA)

First, 96-well plates were coated with 50 µL (1 μg/ml) of PFO (MyBioSource, San Diego, CA) or SLO (Bio Academia, Osaka, Japan). The plates were washed four times with 0.1% Tween20 in sterile PBS (PBS-T), and then blocked with 1% BSA in PBS-T for 2 h at room temperature; the diluted HS1, HS2, and HS3 were incubated for 2 h at room temperature. After the samples were washed with PBS-T, they were incubated with anti-mouse IgG conjugated to horseradish peroxidase (Southern Biotech, Birmingham, AL) for 2 h at room temperature. After washing the samples with PBS-T, 0.5 mg/ml *ο*-Phenylenediamine dihydrochloride (Sigma-Aldrich) in 0.05 M citrate–phosphate buffer (pH 5.0) was used for detection. Absorbance was read at a wavelength of 490 nm with an iMark Microplate Reader (Bio-Rad, Hercules, CA).

#### Immunoblotting

Equal amount (1 μg/ml) of PFO (MyBioSource, San Diego, CA) or SLO (Bio Academia, Osaka, Japan) was suspended in SDS sample buffer (Bio-Rad), and boiled for 5 min. The proteins (10 ng/10 μL) were separated by SDS-PAGE, and then transferred to polyvinylidene fluoride membranes (Millipore, Bedford, MA). Membranes were then probed with HS1, HS2, and HS3 mAbs at a concentration of 1 μg/ml as the primary antibody. Subsequently, membranes were labeled with anti-mouse IgG conjugated to horseradish peroxidase (GE Healthcare, Buckinghamshire, UK), and visualized using the ECL Western Blotting Detection System (GE Healthcare).

#### Bacterial strains

The invasive strains NIH34 (*emm3* genotype) and NIH230 (*emm49* genotype) were isolated by the Working Group for Beta-hemolytic Streptococci in Japan [16] from a patient with STSS, diagnosed as per the criteria proposed by the Working Group on Severe Streptococcal Infections [27]. The culture and preparation of bacteria were performed as previously described [28].

#### Human neutrophil killing assay

Human neutrophils were isolated from the venous blood of two healthy volunteers, in accordance with a protocol approved by the Institutional Review Board for Human Subjects, National Institute of Infectious Diseases, Japan (Permit number: 756). This study complies with the guidelines of the Declaration of Helsinki. A modified chemotaxis assay was performed as previously described [15, 16]. Briefly, 3 × 10^5^ neutrophils in Roswell Park Memorial Institute medium containing 25 mM HEPES and 1% FBS were cultured on Transwell inserts (3 μm pore size, Coaster, Corning, NY) in 24-well plates containing 600 µl medium, or 100 nM interleukin (IL)-8 solution (Peprtec, London, UK); these were incubated with or without 3 × 10^6^ bacteria in the absence or presence of HS1, HS2, HS3 mAbs, and control mouse IgG (Rockland, Gilbertsville, PA) for 60 min at 37 °C prior to the assay. After 60 min incubation, cells in the lower wells were collected and 10^4^ 10 μm microsphere beads (Polysciences Inc., Warrington, MA) were added. Cells were stained with propidium iodine (Sigma-Aldrich) for flow cytometry to quantify viable neutrophils, and were analyzed using a FACSCalibur (Becton, Dickinson and Company).

#### GAS infection in a mouse model

All animal protocols were approved by the Animal Experiments Committee at the National Institute of Infectious Diseases (Permit numbers: 116044, 118014), and were compliant with the Guide for Animal Experiments Performed at the National Institute of Infectious Diseases, Japan. Six-week old C57BL/6 male mice, 20–25 g, were purchased from Japan SLC (Shizuoka, Japan), and maintained in specific pathogen-free conditions. Mice were kept in collective cages (5–6 mice/cage) under a 12-h light/dark cycle and received water and feed ad libitum. GAS isolate (4.0 × 10^7^ colony-forming units in 0.5 ml PBS) was inoculated intraperitoneally into 6-week old C57BL/6 male mice (5 or 6 mice per group). 1 h before or after infection, mice were administered an intraperitoneal injection of HS1, HS2, HS3 mAbs, and control mouse IgG (Rockland) (1 mg per mouse). Mouse survival was monitored daily for the indicated time period. At the end of the experiment and the humane endpoint, mice were euthanized by cervical dislocation under anesthesia. The humane endpoint was applied when abnormal appearance over a prolonged period with no visible indications of recovery was observed.

#### Statistical analysis

Data are expressed as mean ± standard deviation (SD). Statistical analyses were performed using GraphPad Prism 8 (GraphPad Software, San Diego, CA). Statistical significance was determined by the log-rank test. A value of p < 0.05 was considered statistically significant. *p < 0.05. The number of animals used in each experiment was determined on the basis of previously obtained results with the experimental model system [21, 28]. Figure legends indicate the number of independent experiments and biological replicates (n) used for statistical analysis.

### Results

#### Cross-reactivity of anti-PLO mAbs with SLO

**Fig. 1:**
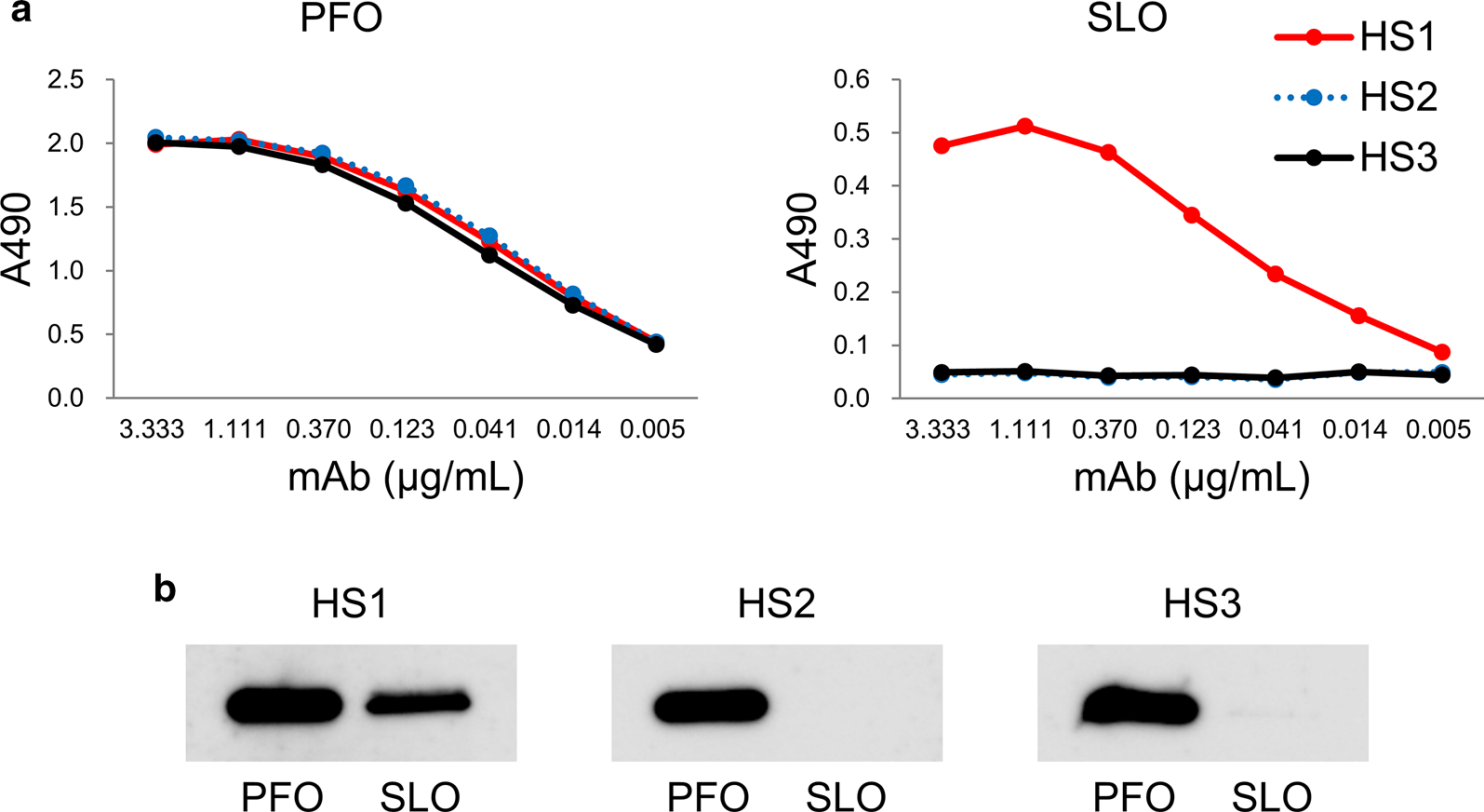
HS1 mAb bound to SLO. **a** ELISA results of HS1, HS2, and HS3 mAbs binding to PFO and SLO. **b** Immunoblotting analysis of HS1, HS2, and HS3 mAbs binding to PFO and SLO

Hybridomas that produce neutralizing mAbs 3H10, 4D8, and 2C5 against PFO [26] were re-cloned and renamed HS1, HS2, and HS3, respectively. We examined cross-reactivity of these mAbs by ELISA (Fig. 1a) and immunoblotting (Fig. 1b and Additional file 1: Fig. S1). HS1 mAb, but not HS2 and HS3 mAbs, was reactive with SLO. These results suggest that HS1 mAb may recognize a certain common epitope or similar structure on both PFO and SLO.

#### The neutralizing mAb HS1 treatment improves the survival rate of neutrophils in the presence of STSS clinical isolates

**Fig. 2:**
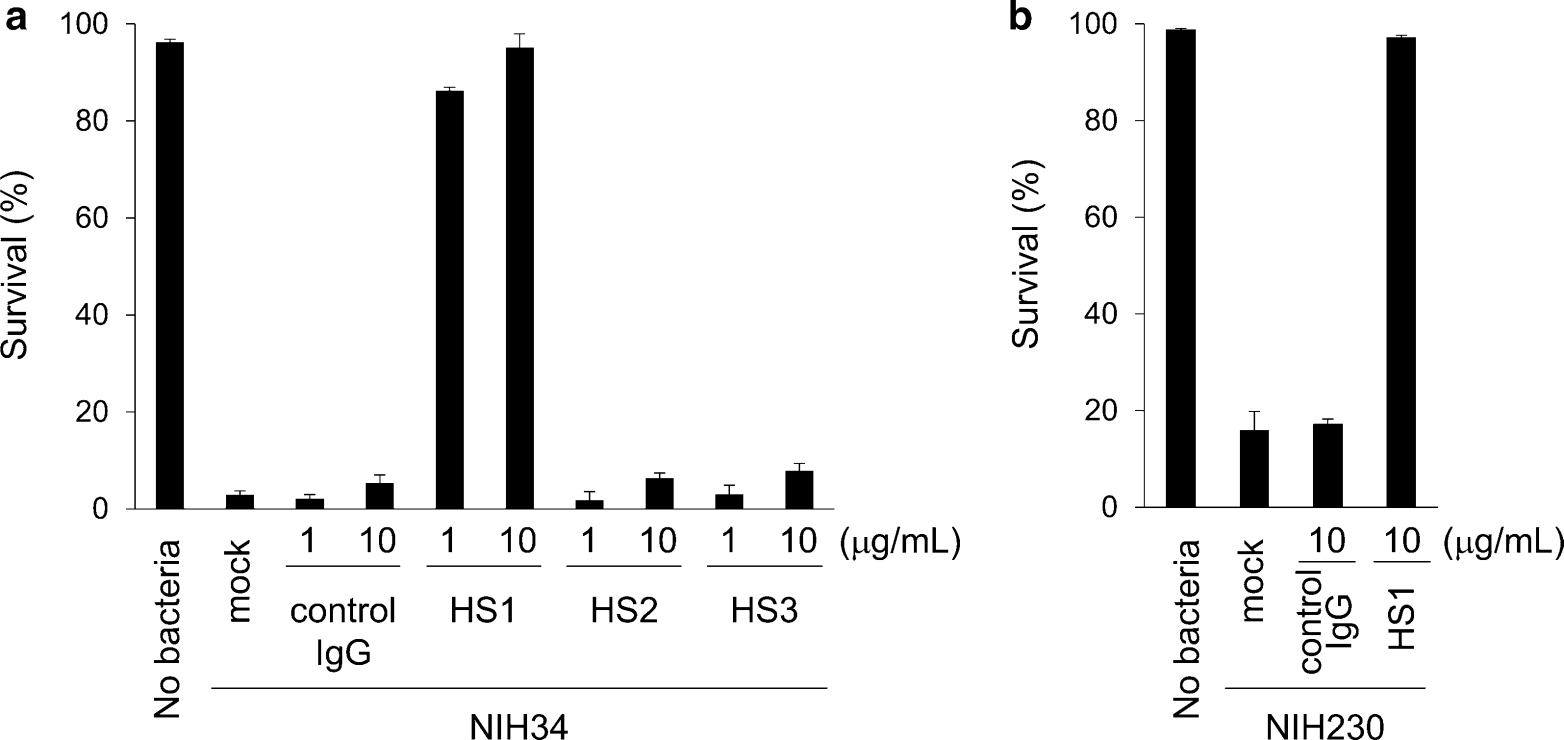
Effects of HS1 mAb treatment on STSS clinical isolate-infected human neutrophils. The results show the proportion of live neutrophils that migrated into lower wells in response to IL-8 alone (no bacteria) or IL-8 in the presence of the STSS clinical isolates (**a**) NIH34 (*emm3*) and (**b**) NIH230 (*emm49*) (3 × 10^6^ colony-forming unites/well) treated with or without 1 μg/ml or 10 μg/ml HS1, HS2, HS3 mAbs, and control mouse IgG. Data are expressed as mean ± SD (n = 3). Data are representative of two independent experiments

We next investigated the neutralizing capacity of HS1, HS2, and HS3 mAbs for the cytotoxic activity of SLO against human neutrophil survival in the presence of an STSS clinical isolate. We have previously established a sensitive in vitro assay for GAS virulence based on SLO activity, by measure of neutrophil viability using a modified chemotaxis assay [15, 16]; human neutrophils that migrate into lower wells in response to IL-8 are killed by the SLO-producing STSS clinical isolates in a contact-dependent manner [15, 16]. Figure 2 shows that killing of human neutrophils by the STSS clinical isolates was inhibited by HS1 mAb. Control IgG, HS2, and HS3 mAbs did not inhibit neutrophil killing by the STSS clinical isolate (Fig. 2a and b). These results suggested that HS1 mAb was acting as a neutralizing mAb against SLO.

#### Pre- and post-treatment with HS1 mAb improves survival of mice infected with an STSS clinical isolate

**Fig. 3:**
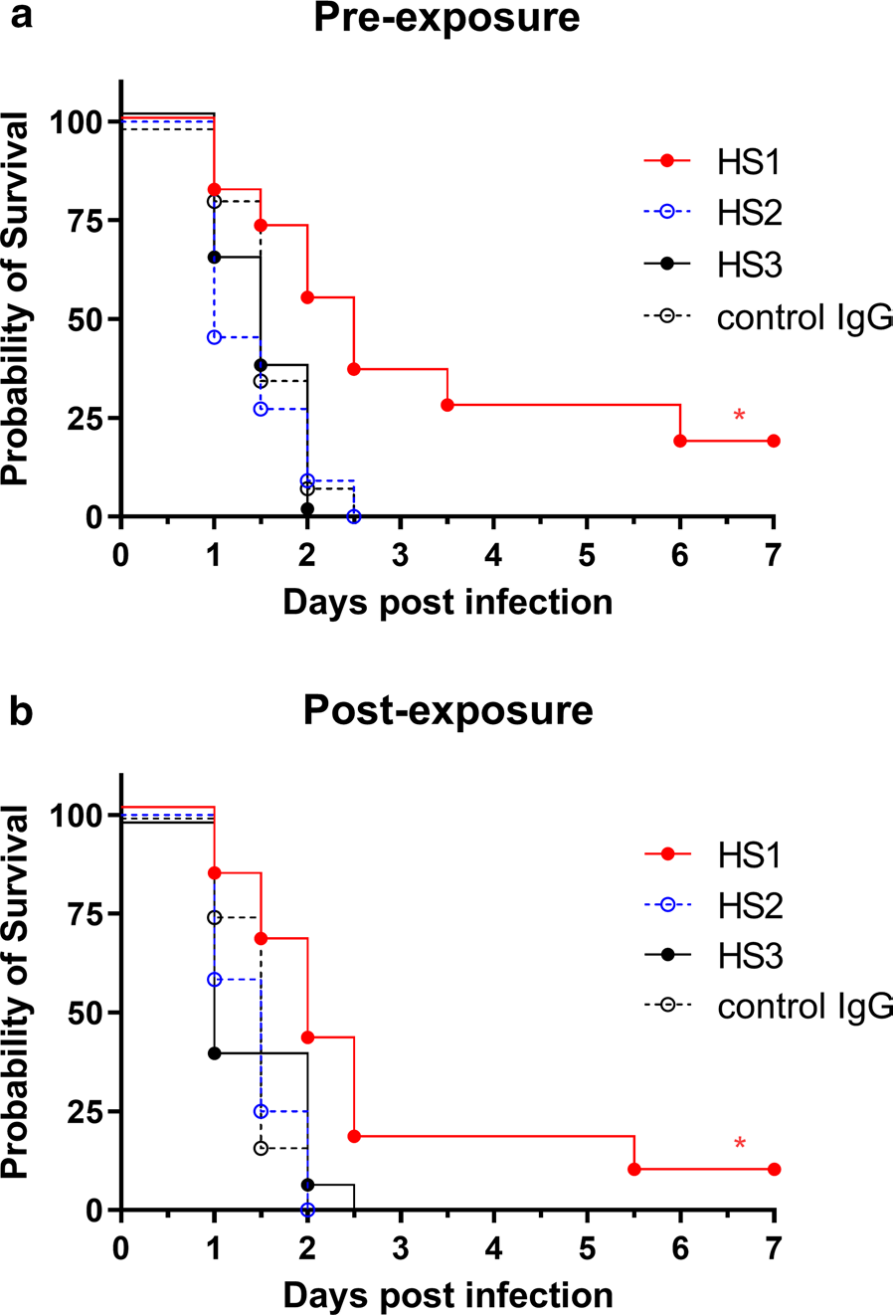
Effect of HS1 mAb administration on STSS clinical isolate-infected mice. C57BL/6 mice (male, 6-week-old) were intraperitoneally inoculated with NIH34 (4 × 10^7^ colony-forming unites per mouse). HS1, HS2, HS3 mAbs, and control mouse IgG (1 mg per mouse) were administrated 1 h **a** before (n = 11 mice per group) or **b** after infection (n = 12 mice per group). Mouse survival was monitored for the indicated time period. The combined data from two independent experiments (n = 5 or 6 mice per group) are shown. *p < 0.05 by log-rank test versus control mouse IgG-treated group

To verify the effect of the neutralizing mAb HS1 on infection in vivo, a 7-day survival rate was undertaken, using a mouse model intraperitoneally infected with an STSS clinical isolate. Pre-treatment with HS1 mAb, but not control IgG, HS2, and HS3 mAbs, significantly improved the survival rate of mice infected with the STSS clinical isolate (Fig. 3a). Furthermore, post-treatment with HS1 mAb also, but not control IgG, HS2, and HS3 mAbs, improved the survival rate of mice infected with the STSS clinical isolate (Fig. 3b).

### Discussion

Recently, high mortality in STSS patients has not been significantly reduced despite adequate antibiotic treatment in high-income countries, and the efficacy of combined antibiotic therapy and adjunctive intravenous immunoglobulin (IVIG) therapy in STSS has been evaluated [29]. A high dosage (0.5–1.0 g/kg) of IVIG is required for IVIG therapy, which is expensive and difficult to achieve. Therefore, the development of targeted therapy for STSS virulence factors is required. We have previously reported that SLO is one of the major virulence factors in STSS [15, 16]. Here, we demonstrated that antibody therapy against SLO may be a new adjunctive treatment for STSS.

In this study, the ability of an STSS clinical isolate to kill neutrophils was inhibited by the addition of HS1 mAb, but not HS2 and HS3 mAbs. Furthermore, in the mouse model, pre- and post-treatment with HS1 mAb, but not HS2 and HS3 mAbs, significantly increased the survival rate of mice infected with an STSS clinical isolate. These results suggest that HS1 mAb, which prevents neutrophil death in GAS infection, may be used as an adjunctive protective antibody for STSS.

SLO belongs to a large family of pore-forming toxins called CDCs [9–13]. CDCs are secreted by gram-positive bacteria, including *Bacillus*, *Listeria*, *Lysinibacillus*, *Paenibacillus*, *Brevibacillus*, *Streptococcus*, *Clostridium*, *Gardnerella*, *Arcanobacterium*, and *Lactobacillus* [30]. Notably, it has been reported that PFO neutralizing mAb 3H10, an original clone of HS1 mAb, has cross-reactivity not only with SLO but also with *Clostridium bifermentans*-derived bifermentolysin and *Clostridium sordellii*-derived sordelliolysin [26, 31]. If HS1 mAb binds to other CDCs and can neutralize them as well as SLO, PFO, bifermentolysin, and sordelliolysin, HS1 mAb may become an effective adjunctive therapeutic antibody for many gram-positive bacterial infections.

## Limitations

We did not identify the specific conformational epitope of SLO and PFO recognized by HS1 mAb. In vivo protection assays were performed using only one strain, single dose, and intraperitoneal route of mAb administration.

## Supplementary information

**Additional file 1.** Immunoblot analysis of HS1, HS2, and HS3 mAbs binding to PFO and SLO.

## Data Availability

Complete sequence data that support the findings of this study have been deposited in the DNA Data Bank of Japan (DDBJ), the EMBL Nucleotide Sequence Database, and GenBank under the following accession numbers (LC545573, LC545574, LC545575, LC545576, LC545577, LC545578). The datasets used and/or analyzed during the current study are available from the corresponding author on reasonable request.

## Abbreviations

CDC: Cholesterol-dependent cytolysin
GAS: Group A *Streptococcus*
mAb: Monoclonal antibody
PFO: Perfringolysin O
SLO: Streptolysin O
STSS: Streptococcal toxic shock syndrome
