# Supplementary figures and images for: An anti-perfringolysin O monoclonal antibody cross-reactive with streptolysin O protects against streptococcal toxic shock syndrome
Source: BMC Res Notes. 2020 Sep 5;13:419. doi: 10.1186/s13104-020-05264-2 (PMC7487723; doi:10.1186/s13104-020-05264-2)

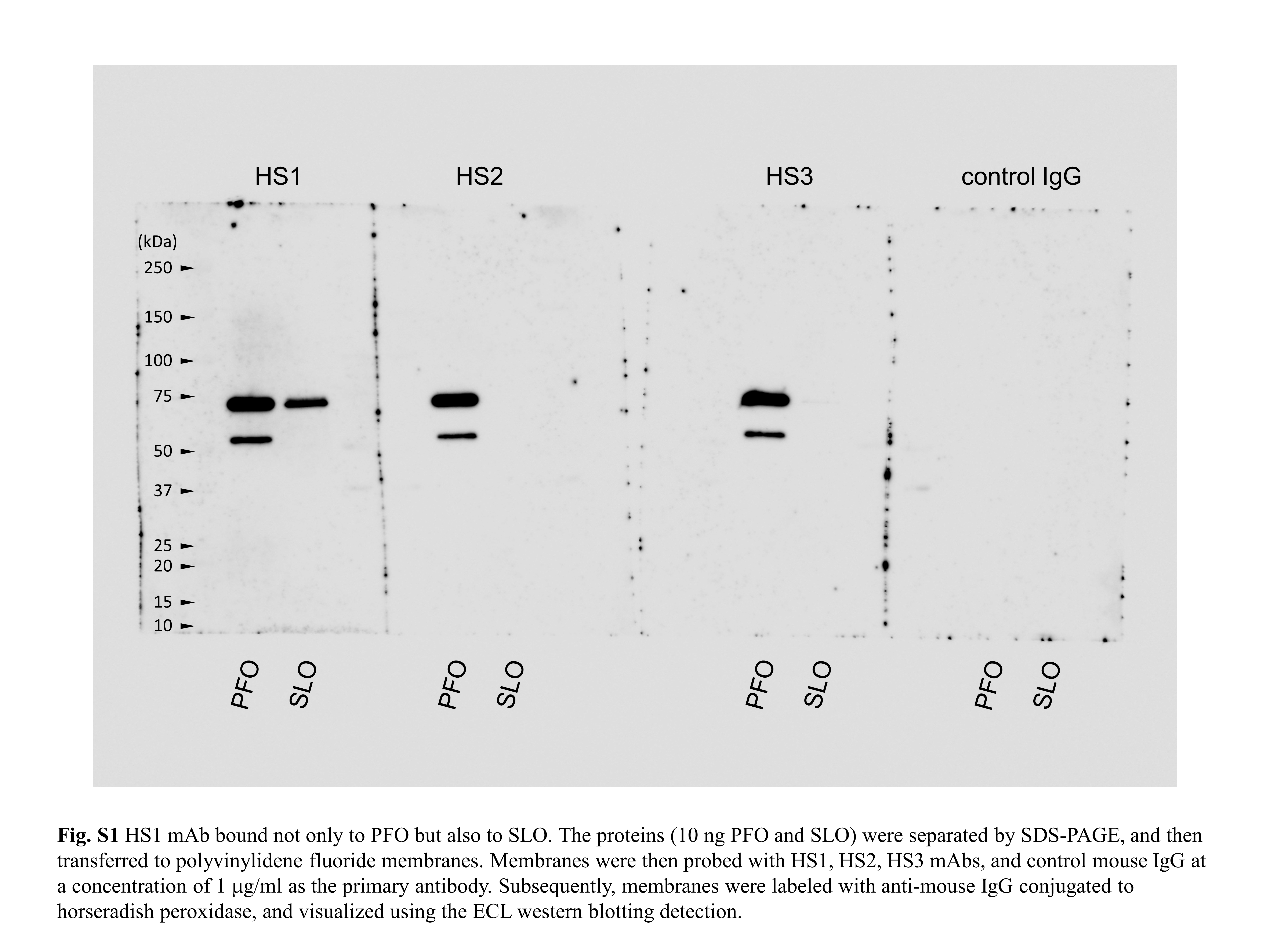

Supplement: Supplementary file 1 — Additional file 1. Immunoblot analysis of HS1, HS2, and HS3 mAbs binding to PFO and SLO. [file 13104_2020_5264_MOESM1_ESM.tif]
